# Recombinant Chromosome 4 from a Familial Pericentric Inversion: Prenatal and Adulthood Wolf-Hirschhorn Phenotypes

**DOI:** 10.1155/2013/306098

**Published:** 2013-05-16

**Authors:** Francesca Malvestiti, Francesco Benedicenti, Simona De Toffol, Sara Chinetti, Adelheid Höller, Beatrice Grimi, Gertrud Fichtel, Monica Braghetto, Cristina Agrati, Eleonora Bonaparte, Federico Maggi, Giuseppe Simoni, Francesca Romana Grati

**Affiliations:** ^1^Research and Development, Cytogenetics and Molecular Biology, TOMA Advanced Biomedical Assays S.p.A., 25/27 Francesco Ferrer Street Varese (VA), 21052 Busto Arsizio, Italy; ^2^Servizio di Consulenza Genetica dell'Alto Adige, Dipartimento di Pediatria, Ospedale di Bolzano, 39100 Bolzano, Italy; ^3^Laboratorio di Citogenetica, Ospedale di Bolzano, 39100 Bolzano, Italy; ^4^Servizio di Anatomia e Istologia Patologica, Ospedale di Bolzano, 39100 Bolzano, Italy; ^5^Reparto di Ostetricia e Ginecologia, Ospedale di Bolzano, 39100 Bolzano, Italy

## Abstract

Pericentric inversion of chromosome 4 can give rise to recombinant chromosomes by duplication or deletion of 4p. We report on a familial case of Wolf-Hirschhorn Syndrome characterized by GTG-banding karyotypes, FISH, and array CGH analysis, caused by a recombinant chromosome 4 with terminal 4p16.3 deletion and terminal 4q35.2 duplication. This is an aneusomy due to a recombination which occurred during the meiosis of heterozygote carrier of cryptic pericentric inversion. We also describe the adulthood and prenatal phenotypes associated with the recombinant chromosome 4.

## 1. Introduction

Wolf-Hirschhorn Syndrome (WHS) results from partial deletion of the distal short arm of chromosome 4 (4p16.3). The clinical features are variable, with increasing severity depending on the extent of the deletion, although the minimal diagnostic criteria should include the association of typical facial appearance, growth delay, mental retardation, and seizures [1]. Considering that small and large 4p16.3 deletions are associated with mild and severe WHS phenotype, respectively, Zollino et al. [1] have suggested a WHS classification in three categories based on the clinical presentation, all sharing the minimal diagnostic criteria: “mild” form (deletions < 3.5 Mb) refers to patients with a mild mental retardation (MR), possible fluent language, and usually independent walking by the age of 2-3 years; “classical” form (deletions 5–18 Mb) is characterized by major malformations, severe psychomotor delay (PMD), delay or absence of speech, and late walking; “severe” one (deletions > 22–25 Mb) has severe PMD and MR, facial anomalies, severe scoliosis, and psychotic behaviour. At a molecular level, two WHS critical regions (WHSCRs) have been identified: the WHSCR region, which is 165 Kb in size and it is located at about 2 Mb from the telomere between the markers D4S166 and D4S3327 [2], and the WHSCR-2 region which embraces a 300–600 Kb interval between the loci D4S3327 and D4S168 and it is mapped distal to WHSCR at about 1,9 Mb from the telomere [1]. Most of the 4p16 deletions involved in WHS occur *de novo*, but in 10%–15% of cases the derivative chromosome 4 originate from chromosomal rearrangements in one of the parents. Four different rearrangements are reported [1]: (1) isolated 4p deletion (70% of patients); (2) unbalanced translocation (22%); (3) inverted duplication associated with terminal 4p deletion (6%); (4) recombinant chromosome 4, rec(4), consisting of unbalanced pericentric inversion with a large 4q segment duplicated on the deleted 4p (2%). Herein we describe a familial WHS caused by a rec(4) with terminal 4p16.3 deletion and terminal 4q35.2 duplication. This is an aneusomy due to a recombination which occurred during the meiosis of heterozygote carrier of cryptic pericentric inversion. 

## 2. Materials and Methods

### 2.1. Clinical Report

A 28-year-old pregnant woman, first child of healthy and nonconsanguineous parents, referred to a genetic counselling at the 14th week of gestations (wg) for the presence of MR in her family history. Her brother, a 23-year-old boy, presented facial dysmorphisms, hypotonia, PMD (independent walk at 24 months), MR, and seizures since the age of 2 years. He was born at the 41st wg by an induced delivery, after an uncomplicated pregnancy (weight, W, 2.5 Kg; height, H, 49 cm; head circumference, HC, 32.5 cm). He also (W 53,8 Kg, H 157,7 cm, HC 50.5 cm) showed pre- and postnatal developmental delay, myopia, lip and tongue tie, malocclusion of teeth, cryptorchidism, social behaviour, and frequent respiratory infections. After prenatal cytogenetics analysis and genetic counselling the pregnancy was terminated at the 20th wg. At autopsy the fetus showed a growth retardation (W, 250 g; H, 23.5 cm; HC, 15.5 cm), facial dysmorphisms (hypertelorism, prominent eyes, low-set ears, beaked nose, and mild micrognathia) and the absence of major external or internal malformations. In addition the weight of placenta was not up to expectation compared to week of gestations (W 85 g, corresponding to the 16th wg), with a single umbilical artery. 

### 2.2. Cytogenetic and Molecular Cytogenetic Analysis

Chromosome analysis was performed in agreement with the Italian Guidelines which are consistent with the European ones. Standard protocols were used to set up the cultures and chromosome preparations; the applied banding techniques were GTG and QFQ; karyotypes were formulated following the ISCN [6]. The other molecular cytogenetic analysis (fluorescence *in situ* hybridization, FISH and array comparative genomic hybridization, aCGH), applied to increase the chromosome level of resolution, was done according to the manufacture's protocol. FISH analysis was performed using TelVysion 4p/4q and LSI WHS/CEP4 probes (Vysis, Des Plaines, USA). Array CGH was carried out using a genome-wide BAC platform (ConstitutionalChip 4.0, PerkinElmer Wallac, Turku, Finland) with a dye-swap approach. The average spatial resolution is 600 Kb and the reference DNAs were commercial pools of human male or female DNAs (Promega Corporation, Madison, Wisconsin, USA). The referred samples were analysed using a dye-swap experiment by reversal dye approach hybridizing the sample DNA against a sex matched human commercial DNA reference (Promega). Data were analysed with the OneClickCGH 4.3.3 Software (PerkinElmer) and positions are according to Human Genome Built 36 (hg18, Assembly Mar 2006).

The aCGH results were always visualized by FISH using selected BAC clones on metaphases and/or on nuclei. BACs derived from RP11-library (PerkinElmer Wallac, Turku, Finland). 

## 3. Results and Discussion

**Figure 1:**
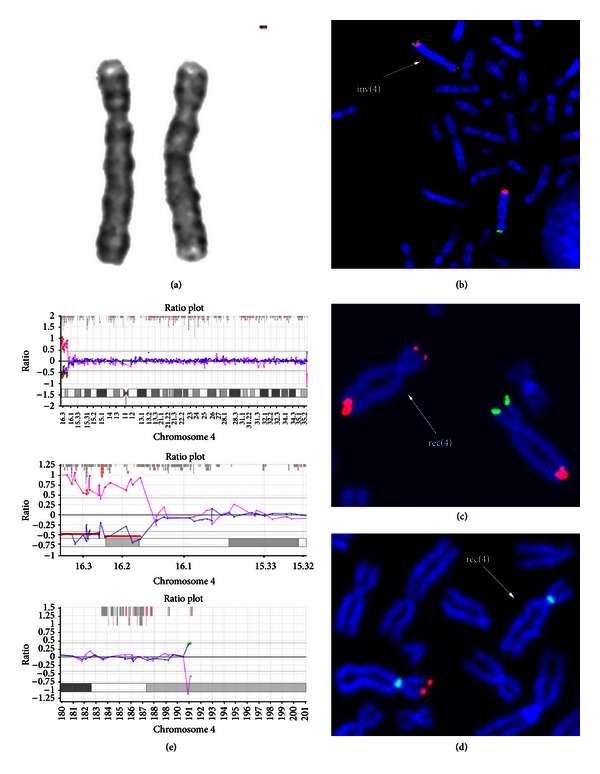
(a) GTG partial karyotype, only chromosomes 4 of heterozygote inversion carrier are shown (b) FISH on lymphocyte metaphases with tel4p and tel4q probes of heterozygote inversion carrier: tel4p probe signal (green) is located on the 4q subtelomeric region, while tel4q probe (red) shows a signal both on the subtelomeric 4q region and on the subtelomeric 4p region (low intensity) (c) FISH on amniocyte metaphase. The rec(4) (indicated by the arrow) shows the duplication of the tel4q probe signal (red) and the lack of the tel4p probe signal (green signal on normal chromosome). (d) WHS specific probe signal (red) is absent on rec(4), while CEP4 control probe signal (green) is shown on both chromosome 4 (e) Array-CGH of chromosome 4.

The karyotype of the pregnant patient, performed with GTG-banding (level of resolution >550 bands, Figure 1(a)), revealed a chromosome 4 with a structural abnormality. To define the rearrangement, FISH was carried out using 4p (tel4p, 4p16.3, locus D4S3359) and 4q (tel4q, 4q35.2, locus D4S2930) subtelomeric specific probes. The tel4p signal was located on 4q, while the tel4q signal was splitted. Namely, two tel4q diminished signals were presented on 4p and on 4q, respectively (Figure 1(b)). This finding demonstrated the presence of a cryptic pericentric inversion of a chromosome 4 with one of the two breakpoints mapped within the region recognized by the tel4q probe [karyotype: 46,XX,inv(4)(p16.3q35.2).ish inv(4)(p16.3)(D4S3359−,D4S2930+)(q35.2)(D4S2930+,D4S-3359+)]. Amniocentesis was performed at 18th wg combining QFQ karyotype with TelVysion 4p/4q and WHS FISH analysis; we identified a partial duplication of tel4q region on 4p and the deletion of both tel4p and WHS critical region. The aberrant chromosome 4 was defined as rec(4) and the fetal karyotype was 46,XX,rec(4)dup(4q)inv(4)(p16.3q35.2)mat. ish rec(4)dup(4q)inv(4)(p16.3)(D4S3359−,WHSC1−,3′D4S29-30+,5′D4S2930−)(q35.2)(D4S3359−,WHSC1−,3′D4S2930+, 5′D4S2930+) (Figures 1(c) and 1(d)). The pregnancy was terminated at 20th wg. Karyotype and FISH analysis with the previously described probes (TelVysion 4p/4q and WHS), extended to the pregnant patient's brother, showed the same rec(4) chromosome observed in the fetus. In addition, array-CGH analysis has been used to characterize the size of both the 4p deletion and the 4q duplication. The size of 4p deletion was 6,293 Mb with breakpoint between RP11-586D19 and RP11-91B20 clones, which are the last retained and the first deleted ones, respectively; 4q duplication was 0,67 Mb in size. RP11-354H17 and RP11-45F23 are the last balanced and the first duplicated clones, respectively. To our knowledge, in the literature are reported only three other familial WHS cases (Table 1) caused by the presence of a rec(4) with partial monosomy of the distal 4p segment and partial trisomy of the distal 4q region, resulting from meiotic recombination in a parent carrier of a pericentric inversion of large size [3–5]. It is well known that a crossing-over within large inverted segment is very likely to take place and, as demonstrated also by the presence of two patients in our family, the genetic risk to heterozygotes for the large inv(4) would be high. In addition we have characterized the deletion and duplication size by aCGH. Specifically, the 4p deleted region, about 6.293 Mb, encompasses (i) the WHS critical regions (WHSCR e WHCRS2), (ii) *LETM1* gene [7], and a 1.9 Mb terminal region, both associated to seizure disorder, (iii) a region between 1.9 and 1.6 Mb from the telomere responsible for facial dysmorphisms and growth delay, and (iv) a region of 2.2 Mb from the telomere associated to microcephaly [1]. Three genes map in the 4q duplicated region, about 0.67 Mb of extension: *FRG1*, encoding a nuclear protein involved in pre-mRNA splicing [8], *TUBB4Q*, a member of the human beta-tubulin supergene family [9], and *FRG2*, potentially involved in myogenesis [10]. All of them are candidate genes for facioscapulohumeral muscular dystrophy (FSHD). Overall, the size of the deleted and the duplicated portion, the genes encompassed and the known karyotype-phenotype correlations concerning the deleted segment, suggest the loss of the 4p distal region as the unique reason of the clinical features of the fetus and the adult patient. Although the extent of the 4p deletion is to be included within the range of “classic” WHS form, the absence of both severe MR and major malformations, the presence of language and the beginning of independent walking at the age of 2, are suggestive of a “mild” WHS syndrome, rather than a “classic” type.

## 4. Conclusion

In conclusion, the adult patient presents the core WHS phenotype, while the fetus, cause of the obvious reasons of some age-dependent clinical signs, shows a phenotype restricted to the presence of dysmorphic features compatible with the syndrome and to intrauterine growth delay. Knowledge of the fetus with WHS is still limited, but the few cases reported, as the discussed one, showed typical craniofacial dysmorphic signs of WHS and severe IUGR (intrauterine growth retardation). 

## Figures and Tables

**Table 1:** Karyotype and phenotype comparison among cases with recombinant chromosome 4.

	Ogle et al., 1996 [3]	Dufke et al., 2000 [4]	Mun et al., 2010 [5]	Present cases
Karyotype	46,XX,rec(4)dup(4q)inv(4) (p15.32q35)mat	46,XX,rec(4)dup(4q)inv(4) (p16.2q35.1)pat	46,XX,rec(4)dup(4q)inv(4) (p16q31.3)pat	46,XX,rec(4)dup(4q)inv(4) (p16.3q35.2)mat

Prenatal phenotype	ND	ND	Pleural effusion and polyhydramnios	Growth retardation, facial dysmorphisms (hypertelorism, prominent eyes, low-set ears, beaked nose, and mild micrognathia), absence of major external or internal malformations

Adulthood phenotype	Wolf-Hirschhorn Syndrome, left hemiplegia, epilepsy, atrophy of the right cerebral hemisphere, dilatation of the right ventricle, small ventricular septal defect, and no speech	Wolf-Hirschhorn Syndrome	No malformation or dysfunction, preauricular skin tag	Facial dysmorphisms, hypotonia, PMD (indipendent walk at 24 months), MR, seizures, pre- and post-natal developmental delay, myopia, lip and tongue tie, malocclusion of teeth, cryptorchidism, social behavior, and frequent respiratory infections

ND: not done.
